# The impact and importance of place on health for young people of Pasifika descent in Queensland, Australia: a qualitative study towards developing meaningful health equity indicators

**DOI:** 10.1186/s12939-019-0978-2

**Published:** 2019-06-03

**Authors:** Jo Durham, Nicola Fa’avale, Andrew Fa’avale, Catrina Ziesman, Eden Malama, Sarai Tafa, Tamaika Taito, Jori Etuale, Mitieli Yaranamua, Ueta Utai, Lisa Schubert

**Affiliations:** 1 University of Queensland, Brisbane, Australia; 2 Queensland University of Technology, Brisbane, Australia

**Keywords:** Health equity, Social determinants, Health indicators, Youth participatory approach, Pasifika, Australia, Place, Talanoa

## Abstract

**Background:**

Health equity is a priority in the global sustainable development agenda. Available health equity indicators often focus on health outcomes, access to healthcare, risk factors and determinants such as income, education, and gender. Less attention has been given to other social determinants, including those related to place and ethnicity. Measures such as income, education, and gender, however, may not provide policy-makers with sufficient information to redress inequities. In this paper, we begin to develop health equity indicators for young Pasifika peoples in Logan, Queensland, Australia. While health data on Pasifika young people in Queensland is scant, available data suggests significant inequalities. The purpose of the study was to develop an understanding of the drivers of these disparities through the lens of the social determinants of health, to create health equity indicators.

**Methods:**

Following meetings with community stakeholders to develop respectful and collaborative partnership processes, we took a youth participatory action research approach. Six peer researchers (3 male, 3 female) were recruited from the Logan area for the project. Following training, the peer researchers undertook 31 qualitative interviews with young Pasifika (16–24 years old). Data was manually analysed, coded and grouped into themes to develop the draft indicators. Interviews used the culturally appropriate Talanoa storytelling approach.

**Results:**

Six key themes were identified from the interviews and were used to develop example indicators related to: spiritual and socio-cultural dimensions, place, access to culturally responsive services, economic and material dimensions and political dimensions. The results demonstrate health inequities experienced by Pasifika populations are strongly linked to place and their economic, social and cultural position.

**Conclusions:**

This study emphasises the need to understand the multiplicity of place-based factors that interact in complex ways to shape health inequities for young Pasifika peoples. It highlights health equity indicators must go beyond healthcare services, outcomes and a limited number of objective determinants, to include a more holistic focus. Starting to measure health and wellbeing via the lens of the social determinants of health will help to identify where policy-makers and programmes can intervene to begin to more adequately address inequities.

## Background

Health equity is “the absence of unfair and avoidable or remediable differences in health among populations or groups defined socially, economically, demographically or geographically” [1]. Health inequities refer to health differences that are “unjust and avoidable differences in health that stem from some form of discrimination or lack of access to certain resources” [2] and are therefore socially produced and mainly preventable [3, 4]. As such, health equity is about the opportunities a person has to achieve good health as opposed to the personal decisions people make about their health [5]. Despite global improvements in health outcomes and increases in life expectancy in many countries, regardless of their level of development, inequities are widening and of increasing policy concern [6, 7]. These inequities manifest in the levels of health risks to which different population groups are exposed and in differential access to its resources [4, 8]. The need to address widening inequities is enshrined in the post-2015 global development agenda and the pledge to “leave no one behind” [9]. In regard to health, it is particularly relevant to the United Nations Sustainable Development Goal (SDGs) Goal 10, which aims to “reduce inequality within and among countries”, and Goal 3, that aims to “ensure healthy lives and promote well-being for all at all ages” [9].

Ongoing measurement, evaluation and reporting are important aspects of promoting health equity since what gets measured often affects whether and how actions occur [3]. Monitoring health equity also helps to track progress, and can serve as a warning system [10]. Many of the available health equity indicators however, focus primarily on access to quality healthcare, risk factors and health outcomes, employment, education, housing and socio-economic position [11, 12]. Furthermore, while many surveys collect data on place of birth, few surveys collect information on ethnicity, aside from for Aboriginal and Torres Strait Islanders. In order to capture health equity itself, measurement indicators need to also focus more specifically and intentionally on other social determinants of health that drive outcomes. These other determinants, include those related to ethnicity, culture and place and the institutional practices and policy decisions made outside the healthcare system [3, 5]. Current measures used in Australia, as in many other places, however, rarely provide fine enough detail to identify intra-city differences, including differences between different ethnic groups or establish health differences along the social gradient [11]. Logan City, where this study took place has, however, used disaggregated indicators for health in the Immunisation Blueprint 2013–2015, which measures childhood immunisation rates by geographical areas and Australian Bureau of Statistics census data to report the socio-economic factors including motor vehicle ownership, income, English language and overcrowding to identify inequities in immunisation. Whilst Logan City council policy documents value equity, community participation, and recognise the importance of the social determinants of health, aside from immunisation rates, these attributes are not measured [13].

The social determinants of health acknowledge that the health status of a population is improved by addressing the social inequities that impinge upon well-being [14]. Indicators need to provide a nuanced picture including reference to social determinants, otherwise there is a risk of generating superfluous and ineffective policy responses by over simplifing  reality. For example, single measures from a cross sectional survey on built and social environmental features can fail to capture changes in equity and the relational effects of inequalities [3]. This is relevant when determining whether certain populations feel safe in the green spaces that are physically accessible to them. Also important is indicators use data that are verifiable and easily accessible and can be shared in an accessible and compelling manner to stakeholders [15, 16].

In a wealthy country such as Australia, measuring the social determinants of health should mean going beyond the minimum requirements for physical health to include people’s ability to meet their full potential, which requires a level of social and economic security for themselves and future generations [1, 17, 18]. This includes access to education and employment. It also includes access to assets to enable meaningful participation in society. It includes their ability to develop a sense of belonging in, and a sense of autonomy over, their own lives [5, 19–22]. While difficult to measure, there is increasing recognition these conditions must be met to acieve full and healthy lives, and there is a need for researchers, practitioners, and policy makers, to make an explicit commitment to understanding the social determinants of health if we are to deliver practical results [23].

In Australia, one population that experiences disproportionately large inequities across the social determinants of health are people of Pasifika descent. For the purposes of this paper, the term ‘Pasifika’ refers to the peoples of Melanesia, Micronesia and Polynesia. It is important to note that these terms all have origins from colonial contact and classification and are contested amongst their communities. Pasifika includes Māori people who are the indigenous Polynesian people of New Zealand [21]. Using the term Pasifika does not disregard the unique and diverse cultures and languages of each island nation but recognises there are sufficient commonalities in beliefs, values, and practices.

## Pasifika in Australia

In recent times, a large proportion of the Pasifika population in Australia migrated via New Zealand through the 1973 Trans-Tasman Travel Arrangement that allows citizens of New Zealand and Australia to freely visit and migrate to the other country. Available figures on the number of people from Pasifika descent in Australia are thought to be under reported as many are documented as New Zealand citizens [24]. According to the 2016 census however, growth in the number of people claiming Pasifika heritage continues both in absolute terms and as a proportion of the total population [25]. For example, those reporting Pasifika ancestry has more than doubled over the past 10 years, and as such, this rapidly growing population warrants further investment in health equity [25].

Pasifika communities in Queensland are well connected through a rich network of cultural, civic, religious and sporting organizations, with a tradition of working collectively. Despite their unique strengths, however, many of those claiming Pasifika heritage work in unskilled or semi–skilled positions and casual job occupations, and are therefore at risk of irregular employment and inadequate working conditions [26, 27]. Pasifika communities for example, are more likely to be unemployed at almost double the rate of the general Australian population [24] While accurate data on ethnicity of people processed through the Australian criminal justice system are sparse, available evidence suggests Pasifika peoples are disproportionately represented [21]. In 2014, Samoan-born prisoners for instance, had the second highest imprisonment rate in Australia [28].

Changes to the Trans-Tasman Travel Arrangement in 2002 and again in 2017, have also negatively affected those who are New Zealand citizens. Unlike other migrants to Australia, New Zealanders are not considered under Australia’s migration programme or able access benefits provided to other migrant populations. This makes it harder for them to access social service support, Higher Education Loan Programs (HELP), permanent residency and thereafter, Australian citizenship [29]. These changes can also negatively impact second and third generation Pasifika peoples whom, as legal residents but neither citizens nor permanent residents, face exclusion and barriers to prevailing social protections. Residency and citizenship requirements are often difficult for people of Pasifika descent to fulfil as the cost of applications are often prohibitive, with almost half of Pacific peoples residing in Australia not citizens [24]. This, in turn, contributes to disparities in the social determinants of health as well as adverse individual and population health problems due to delayed presentation at the primary care level [30]. The proportion of Pasifika people in Australia with a bachelor degree or above for example, is estimated to be three times lower than the general population [24]. Health data on this population in Australia is scant, available data however, suggests low engagement with preventative primary health care and poorer health outcomes compared to the general Australian population [30]. South East Queensland represents settlement of an estimated 102,320 Pasifika peoples, comprising at least 2.4% of its population [21]. South East Queensland presents a rapidly growing, highly urbanised and youthful population of Pacific Islanders with the majority of its population aged between 0 and 24 years [24]. It has been identified by Queensland Health as a priority population, requiring interventions across the social determinants of health if improvements in health are to be achieved [30]. Specifically, young Pasifika peoples (aged 16–24 years old) have several risk factors that make them vulnerable to poor health outcomes, including early sexual debut, unprotected sexual intercourse, high levels of interpersonal violence, excessive alcohol consumption and use of marijuana, low levels of physical activity and sub-optimal health seeking practices [30]. Of concern, suicide rates (including attempted suicide) amongst young people are significantly higher than other ethnic groups in Queensland [30].

This paper develops proposals for place-based health and well-being equity indicators for Pasifika youth (aged 16–24 years old). The focus on young people is an important dimension of the paper with many scholars recognizing that young people are more affected by where they live than other demographic groups [31, 32]. While we recognise Pasifika peoples themselves are diverse and heterogeneous, with each Pasifika nation having its own set of cultural beliefs, customs, languages, values and traditions as in New Zealand, there are also similarities within each Pasifika community in Australia including community and family connectedness, a desire for self-determination, and altruistic service, which have allowed the research team and Pasifika community to cultivate a discussion of health equity from a Pasifika perspective within this paper [33].

### Study setting

The study was undertaken in Logan City, a local government area situated within the south of the Brisbane metropolitan area in South East Queensland, Australia. The city is ethnically diverse with an estimated one quarter of Logan’s residents born overseas. According to the 2016 Census, Samoa was one of the top five places of birth for Logan residents, and in the top four for places of birth for residents’ father or mother [34]. After English, Samoan is the most spoken language in Logan [34]. It is one of the most disadvantaged urban areas in Australia [35, 36] and has the largest Pasifika population (estimated N ≈ 8755) in Queensland [30] representing nearly 19% of the total population of Queensland. In some schools, Pasifika children comprise more than 50% of the student population [37]. Logan has also been identified as one of twenty hotspots of high youth unemployment [38] as well as having areas of entrenched health inequalities and place-based risk conditions that require tailored, evidence-based solutions [35].

## Study design and methods

The design of this study was influenced by Pasifika values including reciprocity and relationships, respect and inclusion. Stage one in developing the health equity indicators involved the lead researchers (JD, LS) engaging with culturally relevant community stakeholders (*N* = 6) working within Pasifika communities in Logan, to develop respectful and collaborative partnership processes within the community. Elders are held in high regard by virtue of age, experience and position in the community, and engaging with community stakeholders initially was culturally respectful. This early engagement also helped in the second stage of recruiting peer researchers as well as helping us to begin to identify the opportunities and barriers young people faced in leading healthy lives. These stakeholders however were not involved in the research design, methods or subsequent draft indicators.

In the second stage, consideration and understanding the views and lived experiences of young Pasifika peoples was critical. Six young Pasifika researchers were initially recruited through job posting on social media outlets and Pasifika community stakeholder listserves, and became integral to the planning, data collection, data analysis and dissemination of the research components. The peer researchers lived in or had experience of living in Logan, Queensland, Australia, and were aged between 18 and 26 years old. Applications were shortlisted (*N* = 6) after review from the research team and community stakeholder partnerships, and while our intent was to recruit four peer researches, all those interviewed were recruited as peer researchers due to interview performance but also to give us a greater spread of ages and backgrounds within the team. There were three female and three male researchers who identified specifically as either Fijian, Tongan or Samoan and the peer researchers were aged between 16 and 26. The peer researcher team was able to provide us with unique insights about the research design, including sampling, data collection and dissemination of the findings [39, 40].

Further to valuing this research methodology focusing on young peoples’ worth, peer researchers were remunerated at the standard university hourly rate for commensurate with their tasks. The peer researchers had no prior experience of organising or participating in a research project and workshops focusing on research methods and ethics. Training included an introduction to and practice with various qualitative techniques in data collection, including in-depth interviews, focus group discussions and walk-along interviews prior to undertaking the research. The peer researchers however felt more comfortable with the in-depth interviews and also felt these would work best with young Pasifika. The peer researchers were also introduced to Pasifika research methodologies and ways of interviewing and more specifically, the Talanoa approach. Talanoa is a Polynesian word broadly meaning ‘to talk or converse’ and emphasises the importance of communication to better understand a person, a situation or an issue [41]. Based on Pasifika worldviews, it is a personal encounter where people share stories their lived experiences and aspirations  [33, 41]. It is almost always undertaken face-to-face using unstructured storytelling to develop relationship between interviewer and interviewee, with the researcher part of the research itself, rather than an objective bystander [33, 42]. It shares some characteristics of phenomenological approaches focussing on what the meaning of lived experience of the phenomenon has participants [42]. It is also similar to narrative approaches to research, particularly in the process used to acquire information, a difference however is that in Talanoa, culture is a fundamental factor [42].

For the peer researchers, Talanoa provided a way of talking things over with participants rather than following a rigid format and was one that they felt was most culturally appropriate for this research. While the peer researchers organised the data collection themselves, the entire team met regularly to de-brief and seek solutions to any challenges during the research process. The training and de-briefing sessions were facilitated by academics and Pasifika community stakeholder partners to genuinely co-design the research process.

## Methods

This study used a culturally appropriate, qualitative approach of Pasifika people’s holistic models of health [43] and research methodologies as well as data collection by peer researchers using in-depth interviews informed by the Talanoa methodology [33, 42] and youth participatory action research. Youth-led participatory action research is an approach to scientific inquiry of young people’s lived experiences is grounded in principles of equity [44, 45]. Young people are seen as collaborators in the methodological process, which aims to identify change knowledge and practices to improve the lives of youth and their communities [45]. The purpose was to give voice to young Pasifika peoples’ lived experiences to develop an understanding of the drivers of health disparities and the social determinants of health in order to develop culturally responsive health equity indicators that will be further tested and refined and used to design programs measure change. While meetings and relationship-building with community stakeholders helped inform the research, as the peer researchers grew in confidence, consistent with youth participatory action research approach, the peer researchers and the research team shared power through an iterative process as the peer researchers took more ownership in shaping and disseminating the research through community forums the research. The data used to draft the indicators presented in this paper comes exclusively from the interviews with young people. This approach helped to ensure young people were positioned as active and informed contributors with young Pasifika people’s voices at the forefront.

The research methods of Talanoa, as well the youth participatory action research approach and peer-to-peer interviewing of people with shared cultural heritage, ensured researchers knew the culture and context in which they were undertaking the interviews and were able engage and behave relevantly. Importantly, the peer researchers and the use of Talanoa achieved a levelling of power between researcher and interviewee, in the ability to acknowledge the common-sense worldviews of participants since they existed in similar age and social levels, in a created cultural space (vā) [42].

## Sampling

The Pasifika community stakeholders whom our research team worked closely with throughout the research process were identified by relationships within the academic community as well as other groups that were referred through a snowball effect once word spread about this research. The Pasifika community stakeholders’ and peer researchers’ personal and affiliated networks (i.e. high schools, universities, churches, social media accounts, etc.) were critical resources in disseminating electronic flyers for the opportunity to interview for this study. The peer researchers also used a chain referral approach to sampling with each peer research inviting a potential participant from their community into the study and asking participants to introduce (with permission) another potential participant to the peer researcher [46]. This method is thought to potentially reduce volunteer bias and under-reporting of socially undesirable behaviours [46]. This method also maximised opportunities for a diverse sample with our recruitment of peer researchers coming from different social networks [46]. Respondents were then given an information sheet to read, and a consent form to sign, before an interview was conducted. In this case, purposive sampling was employed to recruit young Pasifika for this research.

### Data collection

The peer researchers conducted face-to-face interviews (*N* = 30), ranging from 30 to 60 min on average, with Pasifika young people between 17 and 26 years of age who lived, or had lived in Logan, Queensland, Australia. Interviews took place in venues that were convenient for the participants and ensured confidentiality. Following the Talanoa approach, the interviews took the form of a conversation and exchange of ideas or thinking as the peer researchers and participants engaged in social conversation, story sharing and – inevitably – the co-creation of knowledge. According to Vaioleti [36], “Talanoa can only be effective when there is a good relationship between the teller and the listener”. As such the peer researchers began by introducing themselves, including their background and responding to participants questions in a reciprocal relationship, also sharing their experiences. Being of similar age, experiences and ethnicity, the peer researchers were able to quickly establish rapport and empathy with participants, as was observed in an interview when a peer researcher was empathising in dialogue with participant, “we know the struggle.. .we can relate to you”.

The peer researchers worked in pairs for each interview, for safety and for moral support as well as debriefing and feedback to each other. Each peer researcher conducted five interviews as the lead interviewer and five in the role of a support person, with 30 interviews conducted in total. Questions related to the day-to-day experiences of participants and factors that contributed to their well-being. While the peer researchers developed a question guide for the interviews, using the Talanoa approach to conduct interviews also gave the participants the flexibility to focus on issues of concern to them, allowing their voices to be represented as they engaged in learning about the day-to-day experiences from conversations with other young people of Pasifika descent. While peer researchers had developed an interview guide with themes, Talanoa interviews were relatively unstructured, and took the form of a conversation and exchange of ideas or thinking as the peer researchers and participants engaged in social conversation, story sharing and inevitably, co-creation of knowledge. Language used in interviews was defined by the peer researchers and interviewees themselves, and peer researchers were given free license to adapt questions/themes to suit their interview style as well as the ebbs and flows of the moment while interviewing. Interviews were recorded (one participant did not want the interview recorded and written notes were taken instead) with recorded interviews professionally transcribed and checked by the peer researchers.

## Data analysis

Throughout the data collection and analytical process, the research team met regularly to review learnings and data surrounding the lived experiences of young people of Pasifika descent living in Logan. Data was manually analysed by highlighting text on notes, making notes in the margins and ‘memoing’ of thoughts at a given stage in the analytical process and amended based on further reading of the texts to identify factors that participants felt enhanced their well-being and further defined and refined through discussion in workshops with the peer researchers. Abbreviated versions of participant responses were recorded, coded and grouped into themes on paper and subsequently into Excel. Further, the Fonofale model of Pasifika health, which provides a holistic framework of recognising family, physical, spiritual, socioeconomic status, service, and other areas of cultural importance to Pasifika health [43] influenced how our research team pulled themes and meaning from interviewee data and within the themes identified potential indicators.

Through this process of reading, examining and interpreting the data, the analysis moved from a primarily inductive approach to a deductive approach [47, 48]. While the analysis was largely undertaken by the lead researchers (JD, LS, NF) the data were also analysed together with the peer researchers themselves, in order to identify key indicators reflecting areas of importance and meaning regarding their health. At a future phase in our research, we intend to develop these indicators in partnership with Councils and young people who participated in this study (including PRs).

## Ethics

The research was approved by the University of Queensland’s Human Research Ethics Committee. The concept of reciprocity was central to the research process. This was apparent through the sharing of research findings with young people themselves through a community meeting and formal report, as well as with participants of the interviews and multi-level community stakeholders.

## Results

Six key thematic areas of health and well-being were identified from interviews: spiritual dimensions, socio-cultural dimensions, place, access to culturally responsive services, economic and material dimensions and political dimensions. These key areas will be discussed further throughout this section, contextualizing their meaning and providing examples of young Pasifika voices. The young people interviewed were an almost equal mix of male and female (*N* = 16 and 15 respectively), ranged in age from 17 to 24 years of age (17–19 = 11, 20–22 = 13, 23–26 = 7), and most identified being Samoan (*N* = 20) other participants from Tonga, Maori, Fiji, Cook islands, Kirabati, Tuva, Nuie and Tokelau with several associating with more than one ethnicity. They spanned a range of socio-economic experiences, either in school, graduates, drop-outs, university cultural committee members and church group affiliates. In total, 15 of the participants were born in New Zealand, 12 in Australia and the remainder from one of the Pacific Island State named above. Analysis of the data demonstrated participants took a holistic view of health, with spiritual, cultural, social, economic and political processes intersecting in often contradictory ways to influence health and give meaning to Pasifika young people’s lives. These different but shared aspects of well-being provided a way of weaving together the different layers of health and well-being for young Pasifika peoples in Australia. The following section discusses the observations of 30 peer researcher interviews with Pasifika youth.

### Spiritual dimensions of health and well-being

Spirituality and a personal relationship with the Christian God was seen as central to well-being. The importance of spirituality was also linked to relationships and place. Participants explained how the church was “a big part of who I am” and a place where they could access social connections, social support and meet their religious and cultural needs as well as develop leadership skills. Participants discussed how interwoven religion and spirituality were with their culture and their lived experiences associated with their identity as a Pasifika person; that churches were a way of participating in and maintaining cultural heritage and an important aspect of social capital. According to one participant:

*“It’s a certain type of church commitment and this is the world. This is our world. This is of what is most value to us. Even with health, health is in the back. It’s more about your spiritual health; that’s more important.” (PR1_005)*

Another female participant explained how she and her brother moved to Logan because:

*“God called us over to help serve Every Nation Brisbane at the time, and we knew that there was a need. Also, there was a calling to be able to stay there. We took the risk and obeyed God to be able to move over.” (ST_001)*

Another person described how when she was considering suicide her faith stopped her:

*“I went through a massive phase of suicide, and it was like ... pretty much the only reason I never fell through with it is because I wanted to see Dad again. And from my understanding of the Bible, and all the miracles that God had done through my journey that I knew for sure it wasn't some kind of hopes thing, it wasn't some kind of fairy tale. It was legit, there is a God out there who loves and cares. And then my understanding of His word, and I wanted to make sure that I saw my dad in the end, and so that's why I never ended it myself. I just need to wait until it's His time”*

Most participants belonged to Christian-based religions and attended either multicultural churches or churches with a specific Pasifika identity. Church and religion functioned for many of the participants as a source of social support and meaning, reinforcing values that emphasize service and civic responsibility, with several acting as youth leaders within the church. The notion of social reciprocity and “giving back” or “serving” emerged with particular clarity in interviews. It is important to note, however, of several participants mention their parents’ accepted practices of giving monetary contributions to the church could often create financial stress. The financial stress arose when young people themselves are required by familial obligation to donate a piece of their own finances, mainly from part-time jobs. In this way, many participants stated that whatever small personal income they do collect from part-time work or other avenues, is often not their own. Coupled with full-time schooling, this can create overwhelming stress for young people as they are unable to save their own money for future pursuits and they house a fear of disappointing family if they reject giving.

### Socio-cultural dimensions of health and well-being

Participant interviews suggested that cultural engagement and Pasifika peoples’ cultural capital, such as language proficiency, acceptance by the Pasifika community, pride in identity and Pasifika values, were all important aspects having a sense of belonging. Young participants reflected their desire to participate in meaningful connections and reciprocity within the broader Pasifika community. Indeed, almost all of the young participants described themselves as being a Pasifika individual as well as belonging to their specific cultural group (e.g. Māori, Samoan, Tongan, Fijian). Many also described how this ethnic identity was central to who they were and to their health and well-being noted when one participant described that, “I just like the culturalness of being Māori. .. I just enjoy it. I enjoy it” (MI_01). Another person reflected on the unique strengths of Pasifika peoples and their forebears which provided strength and inspiration for facing current challenges:

*“. . . our ancestors navigated the seas without any maps. They used their instincts. They used the stars. They used the surroundings, but it was the internal spirit of them to pioneer and to relentlessly, fearlessly, courageously go forward for our people.” (PR1_005)*

One young person was proud to be Samoan because it was how god created her:

*“I'm proud to be who I am, because I believe that's who God created me to be, a Samoan” (PR2_ 004)*

A young female participant explained:

*“ … when people ask me where I'm from, I like immediately I say I'm from Samoa – Just because that's my blood. But then people are like, “No, you were born here, you're Australian.” So, I usually kind of identify myself as a Samoan who was born here.” (PR3_002)*

While many participants identified with one or more ethnic groups, as one person said, *“I’m Tuvaluan/Fijian”* and another “*white/Maori*”, while another said she was “Um, an Islander. I ... well, I’m Norwayan, Cook Island, Maori” they tended to mix in groups with people of different backgrounds, with one person friendship groups were:

*“Samoans, Tongans, Tokelauans and some were from New Zealand. Some of my friends were from New Zealand” (PR3_003)*

While a normative and positive way of defining themselves, this also creates potential conflicts in regard to their identity as young Pasifika and the traditional ways of their family as they negotiate their place in Australia. Participants talked about shedding their Pasifika identity in some contexts or being “A Poly with Polys and an Aussie with Aussies”. As one second generation Pasifika participant explained:

*“So, ways of being and ways of doing things at home is still more close to the culture. And yet, a majority of my time is spent in school in a different world again. But if it comes close to home, it’s the culture. But there is that mix of here in the education system or in mainstream system, where it’s a whole different philosophy or ways of thinking and being. But for us, that connection is, because we draw from what is really close down, because, I guess, that’s closer to our very existence is the cultural elements. So, even though we come in to the space, we still look different, we feel different and we are different, in a sense. There is always that battle.” (PR1_005)*

While relationships with parents, family and the wider Pasifika community was particularly important for young people, they often also described feeling silenced by their elders. Young people also recognized that their families wanted them to do well but did not always know how to support them as they navigated different worldviews.

The notion of stigma recurred in several participant interviews due to being of Pasifika descent and the ‘othering’ stereotypes of Pasifika peoples that stigmatized them, implicitly or explicitly. Participants talked for example, about being stereotyped as low socio-economic and educational status, school dropouts, in trouble with the police, and street kids. One young person explained:

*“I'll be walking through a shopping centre and an old lady ... I can see someone just grab their handbag, and they're like frightened and think I'm about to do something. I feel like I'm stereotyped to be the bad criminal. They don't know my story. They don't know who I am, and we're not all like that.” (PR2_004)*

Another negative side of culture several participants spoke about was the amount of respect given to elders which often silenced young people’s voices:

*You need to speak directly to the kids in regard to, “Okay, kids, what do youth want?” It's not so much what we want in terms of the older generation. Just remember that the young person is going to be taking up their lane. Yeah, it's all about raising up leaders, raising up ...(PR4_002)*

### Place and health and well-being

Many of the young people interviewed expressed a strong sense of attachment to Logan, describing it as a safe place, a place where they feel *at home*, rich in cultural diversity, where it was fun to socialize due to the presence of friends and relatives, as noted in a participant’s commentary:

*“I love living in Logan City, there's a lot of, a lot of Polynesians definitely in this location, but there's also different residents with different backgrounds, different cultures, which is really amazing.” (PR2_003)*

One of the older participants and the mother of four young children, who identified herself as Samoan, explained how, compared to where she lived before, she loved in Logan because she was able to “hang out” with other mothers, couples, and other parents of a similar age:

*“But here in Logan, since we've been here, with family and with the church, our children have been more exposed to my culture more. So, that's what I love about the community. It's very accessible for the kids.” (PR2_005)*

This facilitated a strong sense of community as well as the construction of a positive self and ethnic image and the pride that comes with belonging. Several also reported membership of a local community group or club and many mentioned being part of a sports team and having lots of opportunities within the neighbourhood to play sport and exercise. Participants also mentioned the importance of learning about language, heritage and culture in developing a sense of identity. Access to schools, churches, dance groups, fast-food outlets and the library were also frequently mentioned services that participants valued.

While many participants expressed strong attachment and bonds to Logan, participants also said they often felt marked by a “blemish of place”. This was expressed as the result of negative stereotypes and pejorative media representations of Logan in which Logan is constructed as unsafe, with high unemployment, substance abuse, delinquency, and poor health due to bad lifestyle choices. As one person explained:

*“. . .the media has painted a picture and portrayed Logan to be the unsafe place, the ghetto in the sense of they see the crime rate and all this kind of stuff, but for me, myself, being an Islander and been living in Logan for so many years, I'm actually proud to say that I'm from Logan, because I feel I'm at home.” (PR2_004)*

A female participant aged 23 who identified as Samoan and had lived in Logan all her life, explained that while Logan was often seen by outsiders as a poor place and potentially unsafe it for her it was a place where she felt safe:

*“I guess a lot of people like to look at it that way like as the poor area. It's actually an area where I would say, would be safe area for most Polynesians. You've got your people who don't work and stuff like that. Also, with that as well, in this specific area in where we are right now, you've got the train station where the people who don't work, the people who like to come out during the day, and just bum around. Yeah, I guess a lot of people like to look at it that way like as the poor area. It's actually an area where I would say, would be safe area for most Polynesians.” (PR4_001)*

When asked if she would ever consider moving from Logan she responded:

*“Even if I was rich, I would never leave. Logan's always been home. It's just like if I was to live in I don't know, New Zealand. New Zealand will always be home, but Australia is always home, and Logan is always home” (PR4_001)*

Several people talked about how this negative serotyping impacted on them when they went outside of Logan, or how on leaving Logan “you sort of have to shed your Logan skin”. While this developed an attachment to Logan, it also potentially inhibited wider geographical and social engagement. Those who were able to go to university on the other hand, were able to access a wider and more diverse set of social relationships. One female participant described the reaction of university peers on what she had accomplished in terms of education, when she said she came from Logan. Another explained:

*“. . .sometimes when I tell them I'm from Logan, they make fun. Like, you know, people say Logan Bogan.” (PR3_003)*

The above quote helps to highlight the specific expectations or stereotypes associated with coming from Logan and the perceived differences between *we* (i.e. the people who study at a perceived high-status university) and the kind of people [*they* are] who come from Logan.

Another explained how she did not usually reveal her address at university or invite university friends to visit her. In this way, some participants who were at university used strategies of stigma avoidance by attempting to distance themselves from Logan due to the expectation of being rejected for their ties to Logan itself. Others also noted that since being in university, they did not engage in much socializing with people from Logan, with university providing opportunities to explore beyond their home territory and extended their social networks. Despite experiencing stigma related to place, few expressed the desire to move out of Logan, expressing solidarity and strong ties to Logan. In this way, while many of the participants identify strongly with their Pasifika identity and culture, they also distanced themselves from it in some settings.

### Access to culturally responsive services

The need for access to culturally responsive services was mentioned both by community stakeholders and young people, with several noting people often did not use available services because they did not meet their cultural or spiritual needs. Regarding primary and secondary education in Queensland, children of Pasifika descent are entitled to attend primary and secondary school.

Participants expressed a mixture of reactions to their overall school experiences, ranging from “loved it” to “a bit tough.” Given participants rarely talked about learning or school related activities, their experiences were more often shaped by how they were treated at school. Similarly, one young person explained the importance of developing meaningful relationships based on an understanding of the young person’s worldview and ways of being:

*“Like teachers, they say, ‘Oh, there’s not enough teachers, and well, we just have to train the teachers,’ and it’s not about having more Pasifika teachers, but mainstream teachers understanding and being able to build the relationships . . . [the] underlying value would be just the curiosity about who we are and not bringing the knowledge that they get from their academy and imposing it – asking questions about our ways of thinking and our ways of being – because otherwise it’s just one way . . . Okay, so I say, ‘How can I work in with what you know and how you see the world?’ and often that’s why – because it’s like you’re sitting there thinking oh, okay, fix me. You think you’re going to fix me? . . . And so, it’s not – and a lot of us are good at playing that game; it’s like that. . . like, okay, yeah, yeah, and then go away and . . . yeah, there’s no change.” (KI_001)*

One female participant who was in her last year of high school noted that for some who found school really hard and could not engage with the teachers often dropped out, with negative consequences including drug abuse:

*“So, recently, past few years I've noticed that my friends, they're also Pacific Islanders, that they've dropped out of school and they've become your ordinary city bums. So, city bums, what they do is they roam around Logan, they roam around the city and they're taking drugs, alcohol, stuff that mess up with your head. And they're all like 14, 15” (TT_003)*

Few services were reported to be available for these young people apart from some services provided by the church but often the church fund it hard to engage with these people with many reported to end up being engaged in the juvenile justice system, where again they found limited cultural support.

In different ways, the lack of cultural concordance between young Pasifika peoples and mainstream services can lead to their feeling misunderstood, and that existing services do not meet their needs. On the other hand, more culturally respectful services could achieve good results. One participant for example noted how an education program for teenage mothers enabled one young mother she knew to complete her education and subsequently go on to complete an undergraduate degree at university. Several participants also mentioned a teacher at school who while not of Pasifika descent, was able to demonstrate understanding and empathy. Another young female participant of 18 years old who was not doing well at school explained how she went to a multi-cultural service after school to get additional help.

Poor mental health and lack of services was brought up in several interviews often linked to the elder’s views of mental health and the church. As one 18-year-old explained:

*“that's one thing that's real hard on a lot of Polynesian kids that there's no understanding from their parents ... It's something that I feel like they don't believe in it. To try to explain it. That's the one thing … I think from their upbringing, it never really existed, so they think that we shouldn't believe in it too. If you believe in God as well, then that {poor mental health] shouldn't be existing in your life as well. Which makes it quite hard and it gives them a reason to keep sweeping it away. I know that a Pacific Islander [we're one of the highest rates of suicide]” (PR5_001)*

Nevertheless, there were some classes that were offered free and usually provided by local cultural associations and several people thought there should be more of these, especially as some people were disengaging from their cultural heritage:

*“It's called Language, new language learning your own heritage and cultural backgrounds, and that's a class that I'm taking which is a Samoan language class, that happens once a week and it's for free, so they have different ranges of groups, so beginners, intermediate and advanced, and that's something that should be emphasised is the cultural and learning our language. The generation now, we're lacking in our own language, in our cultural language, and also actually in the knowledge of our heritage and where we're coming from, and so these classes are very ... if we had more of them, and free. Everything to do with that –” (PR6_003)*

This participant also felt connecting to her identity would also improve her mental health and, while she identified as Pasifika she also wanted to represent herself as a Tongan-Samoan woman, yet, felt she could not do this as she had no knowledge of the language or cultural. As with many other participants, she felt incorporating aspects of culture into the school curriculum would be a positive thing.

### Economic and material dimensions of health and well-being

As well as exposure to social and cultural resources, young people need access to material and economic resources. Priority concerns included higher education, employment and ability to pay bills, as well as affordable access to appropriate goods and services, such as nutritious food. Access to safe housing was also identified as an issue with one homeless person explaining that he lived in an abandoned house with four other people and a few others dropping in and out.

In talking about young parents, one young person explained how:

*“It's difficult to access good foods, healthy food, they just can't afford it because they're trying to pay the bills, so because of that they don't eat well; lots of difficulties with healthy eating, they don't have multivitamins, but a lot of those things they don’t access. I have a lot that have to see the psychologist because they just have so much anxiety; it's just from stress, and depression from stress. . . So they just need help with how can I get this and feed my family cheaply.” (KI_003)*

Added financial pressure included expectations to provide for family members back in their home country, for the church, as well as supporting one’s family in Logan. One female participant of 18 years old explained how like many other people she knew financial issues were critical:

*“Being the middle child, after me it's basically the little kids. So, my role in the family, I work and I pay for their transport to school. I pay for their school fees. The older kids, they pay for rent, they pay for our savings and stuff like that. So, with our family, it's definitely financial.” (PR5_005)*

She also went on to explain that she had been bullied at school and experienced anxiety and depression but using services made her more anxious because f the additional financial strain in her family.

A Tongan-Samoan participant aged 22 years old explained how young Pasifika often drove without a driving license because:

*“As we know, I don't know the percentage, but there's a lot of Polynesians who are driving who don't have a learner's licence, or who are driving on a learners’ licence. That's due to the fact that they probably cannot afford it at the time, or don't have jobs, or their parents just financially not stable at the moment. And so they find other ways to drive.” (P6_003)*

Some people also noted that unemployment was prevalent across the youth population and that job seekers services that would work with them not only on identifying their skills and potential work opportunities, but also developing a sense of comradeship, group motivation and support, would be beneficial for young Pasifika.

### Political dimensions of health and well-being

The political dimensions of health and well-being identified by participants related to access to permanent residency and citizenship, as well as the formal rights associated with this, including access to services such as health care, education, social protection and labour markets. Several participants reported concern around the role of institutional stigma for those holding SCVs (a temporary visa) and not Australian citizenship or permanent residency. For many, the conditions of the SCV made the costs of higher education unaffordable, limiting aspirations and opportunities to work upper labour-market segments.

*“It's hard after school. You study in Australia, and then you have all these things of university. . .but study stops you because you can't afford it. So, you need to work, and by the time you start working, your life is all about that, all about money, you forget, you get stuck in that. And studying just becomes nothing because you're growing in age, and everything just turns around.” (KI_003)*

As another participant explained he also had to look after his family as his older brother was in gaol and unable to get financial help to for higher study, he explained how for him and some of his friends:

*“It's basically if rugby doesn't work out for them then they're going straight to the factory. Their old man knows someone, or their old man's working in the factory so their old man's gonna get them a job there. They don't really go out there and want to be bigger than that.” (PR6_001)*

Being a permanent resident but not able to access social protection services available to other permanent residents, was a common theme as one young single mother explained. While she got some child support, she was not able to access the dole, and she pointed out how this political discrimination also contributed to crimes:

*“Having multiple kids, having huge families and the government doesn't help us with that. They let us suffer. And then they wonder why so many kids go out and steal and as much as I hate to say it, it's a lot of Islanders that go out and they – they fight for food. And they become very violent and they become fairly I guess depressed and brought up. They do a lot of bad things to get money, to get food. They steal.” (TT_002)*

Several of the young people in this study noted how aspirations within young people were constrained by their lack of access to resources:

*“Even going back to Uni, the whole goal of Pacific Islanders, I feel, is to better themselves but they can't better themselves in the education system because there's not much help going out for the New Zealand citizens.” (PR4_004)*

In this way, despite many Pasifika peoples paying taxes, government policy reinforces an “insider-outsider” distinction, imposing specific constraints on their choices and positions in socioeconomic and power hierarchies. For young Pasifika born in New Zealand, this form of discrimination and disparities in the social determinants of health and health outcomes is systematic rather than accidental.

Furthermore, accurate data on Pasifika health and participation in higher education and labour markets is scant with many often documented as New Zealanders. This renders people invisible in datasets and reports and is a form of institutionalized discrimination. Government policies also and leave them disenfranchised from higher education and employment opportunities this would bring, limiting their aspirations and field of influence.

## Discussion

Within this research, participants’ understandings of health and well-being were reflective of Pasifika models of health that include family, beliefs and cultural values and the spiritual, physical, mental and other aspects of life that form the connections between family and culture, often represented metaphorically by Pulotu-Endemann’s Fonofale model [49]. The study suggests that Pasifika young people feel positive about their membership within Pasifika groups. Quantitative surveys in New Zealand have also shown that Pasifika youth typically report high levels of ethnic pride [50, 51]. Membership of the broader Pasifika community also enabled participants to construct a positive ethnic identity, affirm a sense of self, and forge supportive networks in an otherwise marginalizing social structure.

The Fonofale model study underscores how individuals experience the places in which they live, and their importance in shaping young Pasifika health and well-being, as well as their perceptions of the opportunities open to them [52]. Participants in our research, for example, expressed a strong emotional attachment to Logan and the diverse communities that help to define Logan. They expressed feeling safe in Logan, and often, attachment to place is closely associated with feelings and perceptions of safety [53]. Positive affirmation of ethnic identity and attachment to place can be health protective and serve the motivational need to belong. It can also build social capital which can also be protective of health and act as a buffer to some of the negative effects of low socioeconomic status on health [54–56]. The type of social capital revealed in this study related to what has been called *bonding capital* and relates to the close, reciprocal relationships, between family and associations based on a shared social identity [56]. While important, the potential of these networks in linking young people to social capital resources outside of their more immediate family and place-based networks was confined within their specific cultural or place-based networks. Furthermore, in some cases these ties were potentially detrimental to health and placed financial stress on some people. A similar finding was observed in a study of Aboriginal Australians living in urban areas [57]. For those who were at university however, there was evidence that they were being linked to richer social networks both within the broader Pasifika community as well as bridging ties to non-Pasifika peoples.

This study also revealed the presence of stigma at both the individual and institutional level, including, spatial stigma, and the Australian citizenship processes that acts as a barrier to the Pasifika community who hold New Zealand citizenship. Under the government policy, New Zealanders deserving permanent residency and citizenship are those who have the resources to manage the administrative hurdles, bureaucratic complexities and high costs, making it beyond the reach of many Pasifika families. In this way, migration policy, helps determine different levels of power, wealth, access to certain services or prestige due to their migration status positions is an important issue for health equity [58].

Participants, also often mentioned how being of Pasifika descent and/or from Logan, were often defined by their deficiencies, such as, “low socio-economic status” and “low educational attainment” and their health risk factors as “obese” and “smokers”. Such negative labelling can lead people to respond with passive acceptance, acting as barriers to socio-economic, political, and cultural capital outside of the immediate community and reducing civic participation and sense of belonging [59, 60]. On the other hand, many of the participants in this study managed stigma through emphasizing the positive aspects of their culture and Logan.

Access to culturally responsive education and tertiary education, access to transport, spaces for playing sport and celebrating culture, social capital, positive employment arrangements and basic income security as important to young Pasifika peoples’ health and well-being and have been documented as lacking for this population. A common theme was that services, especially for mental health, were not culturally responsive to the needs of young Pasifika and did not always support and strengthen the relational aspects of care. One way of addressing this is to look at ways in which young Pasifika peoples can be directly involved in the conception, planning and delivery of services rather than simply receiving services that have not been designed with their input. Placing service-users as ‘experts’ has been found elsewhere to improve use of services, improve service effectiveness and efficiency and help reduce risk factors [61–63]. Such an approach would also recognize the diverse capabilities and talents of young Pasifika peoples and would allow them to engage in collaborative, rather than paternalistic, relationships with service users, while contributing to health equity.

This study highlights how health equity indicators need to go beyond healthcare services and prevention, to include a focus on the social determinants of health. It emphasizes the need to understand the multiplicity of place-based factors that interact in complex ways to shape health inequities. This includes understanding the dynamic interactions between the material features of place as well as its psychosocial elements, including the meanings people ascribe to places, and how political institutions shape the environments [16, 64]. As the places where people live are constructed not only by their material and physical aspects but also by how people experiences and interact with these aspects, research and health equity indicators need to account for the context-specific differences in social processes, such as power, and cultural expression, and the ways people interact with their environments. This view of health means urban health equity indicators need to move beyond measuring health outcomes or features of the built environment, such as amount of bicycle paths, to understanding the economic, social, environmental, political and cultural dimensions that drive well-being [16, 65].

Monitoring the social determinants of health and well-being can also help to identify which of the determinants are the most responsible for producing inequities in populations of Pasifika young people over time, thereby creating entry points for interventions [4, 5, 22, 66]. This research suggests policy makers and service providers need to ensure policies and programs support health equity through a focus on policies to promote equal opportunities for all people residing in Australia, promote social inclusion, and address the individual and systemic discrimination with interventions aimed at the media, school children, universities, healthcare professionals, medical students and employers. This study also suggests common measures of health and well-being used in Australia are missing important information related to the factors that shape young Pasifika people’s health and well-being, and particularly the impact of place on health, and political decisions that limit access to basic services available to other residents. To redress inequities experienced by Pasifika peoples, we suggest health equity indicators related to the key themes revealed by our research. These include the spiritual, socio-cultural, place, material and economic and political dimensions of health and well-being, with examples of potential indicators listed in Table 1.

**Table 1:** Examples of health equity indicators for Pasifika young peoples

Dimensions	Example indicators
Spiritual	Participation in spiritual or religious activities
	Participation in voluntary service to the community
Socio-cultural / Culturally-responsive	Participation in cultural practices and celebrations
	Connection to culture and sense of identity and self-efficacy
	Feeling of trust and respect
	Membership of community organisations
	Participation in policy and service design
	Culturally responsive preventative health and health promotion, programs and practices
	Use of preventative health and health promotion services
Place and space	Sense of belonging to community
	Sense of safety in community
	Free of experiences of discrimination and/or racism
	Access to community facilities and services (e.g. libraries, formal child care, public transport, recreation)
	Reputation of place and representations in media
Material and economic	Participation in higher education, employment
	Support and assistance for further study
	Financial literacy and ability to save
	Meaningful labour force participation
	Satisfaction with standard of living
	Access to fresh food, and food security
	Access to safe housing
Political	Support in accessing higher education, social protection and labour markets
	Support and assistance for further study
	Policies that promote health across sectors and promote socio-economic and cultural inclusion

While we have presented research from literature and interviews to support the above-mentioned indicators, they require further development via co-design efforts with key participants, representative of all those involved from design to service delivery, to be operationalized. Furthermore, in partnership with Pasifika communities and local councils, it would be of value to select priority measures that represent the larger equity issue being addressed. The intent of these indicators is to generate a set of measures that can be combined to monitor progress against the drivers of health inequity. Ideally, indicators should be able to be expressed as proportions and rates so that they can be compared across different jurisdictions within South-East Queensland with large Pasifika populations. A challenge however, could be having sufficient data to provide meaningful comparisons between jurisdictions, as well as to examine associates with health and well-being outcomes [67]. Nevertheless, advances in mobile information technology are creating new opportunities for place-based data collection [3]. Capturing this information in respectful and meaningful ways however will require participatory approaches to inquiry that include both quantitative and qualitative data against the indicators, as experienced by people, to understand how these different dimensions are constructed and contested.

The next step of this work will be to refine and pilot the proposed indicators to ensure they are relevant and feasible and meet the needs of both policy-makers and Pasifika young people. We will use the indicators to develop and test hypotheses about how these features of place interact and influence specific aspects of health in Pasifika young people’s lives over time. In working with policy-makers, the intent is also to align our proposed indicators with existing government indicators and to evaluate progress over time, including the effectiveness of policy and programs in promoting health and well-being for this population. Furthermore, the current paucity of available data relating to Pasifika peoples across a range of socio-economic indicators including health, education and labour force participation will assist in increasing visibility of Pasifika young peoples. Through this work we also hope to be able to gain a better understanding of unmet needs. While hard to measure and detect, given the lack of robust data in this respective area, such information would be invaluable in informing policy and programs and closing the socio-economic gap in health outcomes. This could include, for example, participatory mapping of the physical and social place in which people live, and visualization activities to imagine and advocate for the changes needed to promote well-being. In Rio de Janeiro, Brazil, a youth-led digital mapping program implemented in partnership between UNICEF and a Brazilian non- governmental agency generated data from mapping place-based observations as evidence for health promoting interventions and policies [68].

## Limitations

This paper contributes to our understanding of Pasifika young people’s experience of living in Logan and its influence on health and well-being. A limitation, however, is that the study was based on experiences in one city, and we recognize that Pasifika peoples live in other cities in Australia in which diverse experiences of health equity may occur. Piloting and implementing the indicators in other areas with large Pasifika populations may provide a more nuanced understanding of how health equity can be expanded. In addition, the indicators were selected based on the subjective experiences of young Pasifika peoples and while this makes them valid, they are open to interpretation and will be revaluated as we work with local councils and Pasifika young peoples through further research initiatives. Furthermore, the proposed indicators were derived from the interview data which did not specifically ask participants to identify and rank the indicators and therefore require further refinement with young Pasifika and with other stakeholders to ensure it is also feasible to collect data on the final selection of indicators. As such, limitations of insider research are acknowledged, however, this method of research appears to be an important component of youth participatory action research as well as the cultural relevance of the project design itself [44, 69]. Ultimately the test of the indicators will be whether they promote awareness and action by different actors across the system, not just within the health sector [3, 8]. Based on their work in Richmond and Nairobi, however, Corburn and Cohen [3, 8] reported increased participation in non–health care specific sectors following the introduction of indicators that focused on the social determinants of health. Furthermore, while the proposed indicators focus on the social determinants that contribute to inequality, we do not mean to sidestep the importance of measuring and disaggregating by population groups socioeconomic outcomes. This simply cements the need for a comprehensive approach to analysis of inequity. Much of this data is collected by various household surveys and implemented by Federal and State governments but is rarely disaggregated by ethnicity or place.

## Conclusion

While the experiences of Pasifika young peoples living in Australia may differ depending on context, this study is important in demonstrating how health inequities experienced by Pasifika populations are produced, and strongly linked to place, as well as economic, social and cultural position. While we have shown that connection to culture, place and strong bonding capital can be health protective, the study also suggests these can have some negative effects. Many of the participants demonstrated unique strengths and the social and cultural skills to navigate between their Pasifika culture and mainstream culture, however, barriers to health equity were often underpinned by discrimination and unequal access to economic and social opportunities. Overcoming these barriers requires policies to address discrimination and stigma and recognise the strengths of Pasifika peoples. While this requires attitudinal changes within mainstream Australian society and the Australian Government, beginning to measure and provide policy makers with information against the social determinants of health is a first step towards addressing health inequities.

## Abbreviations

HELP: Higher Education Loan Programs
SCV: Special category visa
